# Relationship Between Mental Health and the Education Level in Elderly People: Mediation of Leisure Attitude

**DOI:** 10.3389/fpsyg.2020.00573

**Published:** 2020-04-01

**Authors:** Pedro Belo, Esperanza Navarro-Pardo, Ricardo Pocinho, Pedro Carrana, Cristovao Margarido

**Affiliations:** ^1^R&D Unit, Polytechnic Institute of Setúbal, Setúbal, Portugal; ^2^Department of Developmental and Educational Psychology, Universitat de València, Valencia, Spain; ^3^Department of Social Sciences, School of Education and Social Sciences, Polytechnic Institute of Leiria, Leiria, Portugal; ^4^Department of Engineering and Industrial Management, Coimbra Institute of Engineering, Polytechnic Institute of Coimbra, Coimbra, Portugal

**Keywords:** psychological well-being, education level, leisure attitude, elderly, mediation model

## Abstract

**Purpose:**

The present study intends to explore the influence of education on the mental health of retired people and the mediating role of a leisure attitude in this relationship.

**Design/Methodology:**

The sample was simple random, and a confidentiality agreement was established. The sample was composed of 403 Portuguese participants (37.2% male; 62.8% female). The participants completed the MHI and the LAS.

**Findings:**

The hypothesized mediation model showed that a leisure attitude mediated the association between education and well-being. In addition, higher levels of distress were found in participants with higher levels of education.

**Scientific Contribution:**

Our results suggest that old-aged people with high education and a more positive leisure attitude have a better psychological adjustment concerning well-being. Also, a higher level of education can lead to a better perception of aging changes (physical, life, profession).

## Introduction

Recent studies about the relationship between leisure engagement and well-being indicate a positive effect on health outcomes of older adults (Windle et al., 2010). Indeed, it is commonly accepted that spending leisure time is important to improve the quality of mental health (Silverstein and Parker, 2002). Aging is, very often, a life period associated with stressful events (Vasunilashorn et al., 2013; de Frias and Whyne, 2015; Kao and Chang, 2017). As main stressors in later life, we must consider the death of a significant person (Kao and Chang, 2017), health related concerns (Tak, 2006) or disability and chronic disease (Norris and Murrell, 1990; Kao and Chang, 2017). Leisure helps to promote health and has a positive impact on life quality (Liu and Lou, 2016; Winterbotham and du Preez, 2016; Lambrini et al., 2018). As stated by Strain et al. (2002), leisure engagement in later life awakens an individual’s desire to strive for familiar forms of activity. At this stage, it improves well-being by giving people a sense of purpose and increase social integration (Havighurst, 1961; Silverstein and Parker, 2002; Mannell, 2007; Dupuis and Alzheimer, 2008; Adams et al., 2011; Windle et al., 2010; Gow et al., 2014).

Active aging enables a strong social background and a good cognitive function, which are associated with high involvement in leisure time (Fernández-Mayoralas et al., 2015). Tsai et al. (2014) referred in their research that participation in leisure activity is a consequential determinant for successful aging and leisure activity is positively related to motivation (Wu, 2008) and to a leisure attitude. A leisure attitude is an essential element in the study of leisure experiences, and individuals with a favorable attitude toward leisure get more benefits from that involvement (Orsega-Smith et al., 2007; Lee et al., 2018). A leisure attitude can be understood as the cognition and positive or negative affection for leisure, and the prepared status to face leisure life (Ragheb and Beard, 1982; Bennett, 1998; Tsai et al., 2014). Literature suggests that leisure is also a significant tool in the life change to retirement (Mannell, 2007; Scherger et al., 2011; Nimrod and Janke, 2012; Lee et al., 2018). In the case of retired people, an attitude of positive leisure is related to a better health lifestyle (Tsai et al., 2014), a greater leisure participation (Searle and Iso-Ahola, 1988; Tsai et al., 2014), and low boredom experiences (Iso-Ahola and Weissinger, 1987; Tsai et al., 2014), and people who have a positive attitude concerning leisure achieve a more successful retirement (Carpenter and Patterson, 2004). Leisure contributes to health (Dupuis and Alzheimer, 2008; Lee et al., 2018) and, thus, a leisure attitude is an important determinant of satisfaction in later life (Ragheb and Tate, 1993; Orsega-Smith et al., 2007; Lee et al., 2018) and a valuable key to understand the benefits of leisure (Tsai et al., 2014; Lee et al., 2018).

Previous studies have demonstrated that older adults with an intention to adopt positive behaviors report a positive effect on general life (Sullivan and Sharpe, 2006). Also, cognitive attitudes toward leisure influence behavioral intentions (Sullivan and Sharpe, 2006). In this sense, older adults involved in leisure activities (Scheibe and Cartsensen, 2010) and with a positive leisure attitude (Crandall and Slivken, 1980; Iso-Ahola and Weissinger, 1990) experience high levels of emotional well-being; this can be explained by a structural decline in brain emotion-sensitive areas that selectively impair the processing of negative stimuli and protect against threats to well-being (Lawton, 1994; Cacioppo et al., 1998; Kunzmann et al., 2000). Overall, a favorable leisure attitude contributes, in innumerous ways, to preserve health in later life (Dupuis and Alzheimer, 2008; Chang et al., 2016; Lee et al., 2018).

On the other hand, the impact of education on mental health in later age has been explored and measured by years of formal schooling and serves as a predictor of healthy aging (Kavé et al., 2012). Education may be considered in diverse domains of research, such as life satisfaction, once the education level seems to have a positive impact on the cognitive variables (Kavé et al., 2012; Foverskov et al., 2018). Moreover, the influence of education is assumed as a central factor in enabling social participation and allowing old-aged people to appreciate a positive well-being as they grow older (Weiss, 2005; Boulton-Lewis et al., 2006). With a focus on the relationship between education and mental health, which, in addition to age, are points of influence among older adults (Leitner and Leitner, 2012), several studies have found that higher levels of education had a positive relationship with higher profits, a healthy nutrition, and general physical and mental care (Leitner and Leitner, 2012). According to Netz (1989), higher levels of education among older people seem to provide a more optimistic attitude toward life. This variable refers to being healthy and developing positive interactions among old adults (Fernández-Mayoralas et al., 2015; Villar et al., 2016; Cuenca-Amigo et al., 2017), and it describes the support that education provides (Purdie and Boulton-Lewis, 2003).

The present study intendeds to explore the influence of education on the mental health of old-aged people and the mediating role of a leisure attitude in this relationship. It was expected that higher levels of education would be associated with a better psychological well-being. The indirect effect of a leisure attitude in the relationship between education and mental health was also tested.

## Materials and Methods

The sample was simple random, so each member of the group was equally likely to be chosen (Gravetter and Forzano, 2015). The study followed the ethical principles for research in humans, established in the Declaration of Helsinki in relation to informed consent, confidentiality and procedures. The Direction Boards of all the institutions approved the questionnaire and data collection. Participants received information about the research aims (doctoral study) before giving their consent to answer the questionnaire; they were also informed that they had the option to drop out the study at any time without giving any explanation. Data were processed as confidential and anonymized.

Each questionnaire included standard instructions and participants were asked to respond according to the option they felt was the most relevant to them. It was clear that there were no right and wrong answers. After completing the set of questions, they were asked to return the questionnaire. The administration of the questionnaire lasted, on average, 25 min.

### Data Collection

The sample was collected from a convenience sampling in which subjects were at least 50 years old (Power et al., 2018) and retired, living in institutions in North and Center Regions of Portugal. A total of 620 questionnaires were collected, however, only 403 were taken into account (valid responses). The participants (internal or daily users) were asked to answer the questionnaire by the institution director and a multidisciplinary team. It was our intention to spend the least amount of time possible in data collection, due to the physical and psychological characteristics of participants.

This study included 403 participants between 53 and 93 years old, with an average age of 72.9 years old (SD = 8.43). The participants were split into two sub-samples. Data included 150 men (37.2%) and 253 women (62.8%). 216 (54.5%) participants lived in a rural area, while there were 180 participants (45.5%) from an urban area. In the study, 356 participants indicated that they practiced leisure activities (88.3%), while 47 subjects did not (11.7%). The types of leisure activities were social (*n* = 94; 42.7%), physical (*n* = 82; 37.3%) and cognitive (*n* = 44; 20%). Regarding the state of health perception, 332 subjects considered themselves as independent (82.6%), while 70 participants considered themselves dependent (17.4%), as shown in Table 1. Participants were mostly married (*n* = 191; 47.6%) or widowed (*n* = 144; 35.9%). About the education level, 153 participants had the primary school education level (38.4%), 66 participants were illiterate (16.6%), and 70 had a higher qualification (17.6%). When they were asked about how they evaluate their health status, 43.2% (*n* = 174) indicated that it was “neither good, nor bad,” but 40.7% (*n* = 164) evaluated it as “good.” Participants were mostly living at their own homes (*n* = 357; 88.6%), but 46 subjects were institutionalized (11.4%).

**TABLE 1:** Sociodemographic characteristics (*N* = 403).

		*N*	%
**Gender**	Male	150	37.2
	Female	253	62.8
**Area**	Rural	216	54.5
	Urban	180	45.5
**Health Perception**	Independent	332	82.6
	Dependent	70	17.4
**Leisure Activities (practice)**	Yes	356	88.3
	No	47	11.7
	Social	94	42.7
	Cognitive	44	20
	Physical	82	37.3

### Measures

The questionnaire was composed of three sections: Sociodemographic questions, the MHI-Mental Health Inventory (Ribeiro, 2001) and the LAS-Leisure Attitude Scale (Freire and Fonte, 2007). Demographic variables included Age (Y/N), Gender (M/F), Education level, Relationship status, Religion, Residence area (City/Rural), Autoperception of state of health (Dependent/Independent) and questions about the subjects’ leisure practice (Y/N).

#### MHI – Mental Health Inventory

The Mental Health Inventory (Ribeiro, 2001) is a questionnaire used for evaluating mental health issues such as anxiety, depression, behavioral control, positive affect, and general distress. The Mental Health Inventory includes 38 items in which the participant uses a 5 or 6-point Likert-style response (ex. item 9: “During the last month have you felt depressed?”; item 33: “During the last month have you felt anxious or worried?”; item 34: “During the last month have you felt happy?”). The 38 items are distributed among five scales (Anxiety with 10 items; Depression, with 5 items; Loss of Emotional/Behavioral Control, with 9 items; Positive Affect, with 11 items; Emotional Ties, with 3 items). In turn, these five subscales are grouped into two major sub-scales or dimensions that, respectively, measure Distress and Psychological Well-Being (Distress results from the grouping of the sub-scales of Anxiety, Depression, and Loss of Emotional/Behavioral Control; while Psychological Well-Being results from the combination of sub-scales Positive Affect and Emotional Ties). The total score is the sum of the values of the items that make up each sub-scale. There are some items with reverse quote. This instrument helps measuring overall emotional functioning. Research has shown the existence of a positive (psychological well-being, positive mental health status) and another negative construct (psychological distress, negative mental health status). This type of measure is important when the aim is to evaluate health in general, or in the context of epidemiological studies (e.g., epidemiology of health), or in the evaluation of health outcomes.

#### LAS – Leisure Attitude Scale (Portuguese Version)

The Leisure Attitude Scale (Freire and Fonte, 2007) is composed of 36 items divided into three subscales for the three components of attitude – cognitive, affective, and behavioral (ex. item 1: “Engaging in leisure activities is a good choice to spend time”; item 2: “Leisure activities bring benefits to people and society”; item 9: “Leisure activities help people to relax”). Each subscale contains 12 items, all directed to the positive direction of attitude. Likert is the response system used, which has five levels of related responses to express agreement and disagreement, in which 1 reveals an unfavorable or negative extreme attitude (“disagree”) and 5 is associated to a favorable or positive extreme attitude (“totally agree”). Point 3 corresponds to a neutral level on the direction of the attitude (“neither disagree nor agree”). Higher values (above the neutral point) show more positive attitudes and, on the contrary, lower values (below the neutral point) indicate more negative attitudes toward leisure. Thus, if the concept of attitude based on the three components is of relevance for the study of attitudes in general, it is also relevant in the specific study of attitudes toward leisure since it contributes to the investigation of psycho-social and socio-cognitive aspects of leisure, and to know better and understand the degree and type of involvement of the subjects, and also the forms and processes that underlie the change of attitudes toward leisure and quality of life, in specific groups or contexts. Thus, for each sub-scale the minimum possible total value is 12 and the maximum is 60 (neutral point located at 36). Concerning the total scale, the minimum possible value is 36 and the maximum is 180 (neutral point in the value 108).

### Analysis Plan

A quantitative research was used instead of a qualitative analysis, because its purpose was to report data through the statistical analysis of the facts reported about the leisure attitude role in mediation models. Data were evaluated using the Statistical Package for Social Sciences (IBM – SPSS) software, version 24. Mediation models were tested through PROCESS, a computational tool for path analysis-based mediation analysis and moderation (Hayes, 2012). To identify possible covariates that should be introduced into the mediation model, correlations between sociodemographic variables (Age and Gender) and the mediator and dependent variables were also computed. Parametric tests were used to study the relationship between the variables (R Pearson statistic test). Cohen (1988) guidelines were used to describe and interpret the effect sizes of correlations (i.e., weak for correlations close to 0.10, moderate for those near 0.30, and strong for correlations at 0.50 or higher). To examine the indirect effects, a bootstrap procedure was used to evaluate unconditional indirect effects (PROCESS assumes 5,000 “resamples”) at a confidence level of 0.05 (Cohen, 1988; Hayes, 2012).

## Results

Prior to mediation analyses, correlations were analyzed, in order to determine whether any variable should be introduced in the model as a covariate. Education was significantly correlated with psychological well-being (*R* = 0.19; *p* < 0.001) and moderate correlation was found between education and distress (*R* = 0.26; *p* < 0.001). Additionally, a negative association was found between age and distress (*R* = −0.11; *p* < 0.05). It was found a positive moderate correlation between cognitive leisure activity and psychological well-being (*R* = 0.31; *p* < 0.05). Also a significant relation was found between gender and distress (*R* = 0.11; *p* < 0.05). Therefore, age and gender were introduced as covariates into the regression models predicting psychological well-being and distress.

The effect of the independent variable on the proposed mediator (Path a), the effect of the mediator on the dependent variable partializing out the effect of the independent variable (Path b) and the direct effect of the independent variable on the dependent variable (Path c’) are presented.

### Mediation Model 1

**FIGURE 1:**
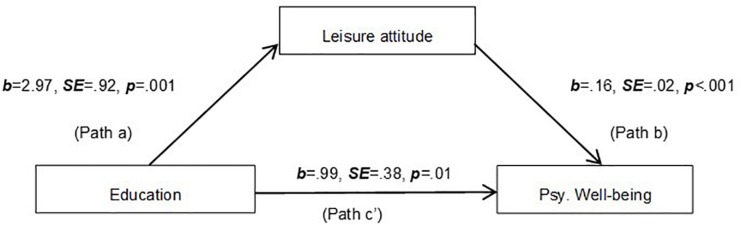
The mediation analysis model including education, leisure attitude, and psychological well-being.

To examine whether a leisure attitude explained the association between education and psychological well-being, a mediation model was tested using a Process macro – model 4 (Preacher and Hayes, 2004). Age and Gender did not covariate at any path, so they were removed and was considered a simple mediation model. Leisure attitude mediated the association between education and psychological well-being. The results indicated that the indirect effect was significant (point estimate = 0.49; 95% BCa CI [0.20; 0.84]) as presented in Figure 1. Education explained 2.76% of the variance of leisure attitude [*F*(1,366) = 10.37; *p* = 0.002]. Education and a leisure attitude explained 16.84% of the variance of the psychological well-being [*F*(2,365) = 36.96; *p* < 0.001], as shown in Table 2.

**TABLE 2:** Model coefficients for the conditional process models presented in Figures 1, 2.

	Direct effect (c’)		Indirect effect (a*b)		R	R-sq	MSE	F
	*b (SE)*	*p*	*b (SE)*	95% Bias-corrected bootstrap CI (LLCI; ULCI)				
**Model 1**	0.99 (0.38)	0.01	0.49 (0.16)	0.20–0.84	0.41	0.1684	162.0407	36.96*
**Model 2**	1.7 (0.48)	0.00	0.32 (0.14)	0.09–0.63	0.31	0.09	250,8681	12.14*

*p < 0.001 SE, standard error; CI, confidence interval, LLCI, lower-limit confidence interval, ULCI, upper-limit confidence interval. All coefficients are unstandardized. 95%BCaCI 95% bias-corrected and accelerated confidence interval.

### Mediation Model 2

**FIGURE 2:**
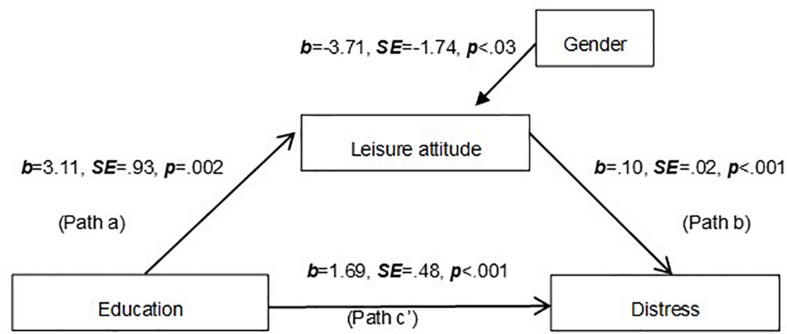
The mediation analysis model including education, leisure attitude, and distress.

To analyze whether a leisure attitude explained the association between education and distress, a second mediation model was tested using a Process macro – model 4 (Preacher and Hayes, 2004). At this Mediation Model only gender was considered as a covariate variable, once this variable influenced the mediator variable. As presented in Figure 2, leisure attitude mediated the association between education and distress. The results indicated that the indirect effect was significant (point estimate = 0.32; 95% BCa CI [0.09;0.63]). Education explained 3.5% of the variance of a leisure attitude [*F*(2,353) = 6.40; *p* = 0.002]. Education and a leisure attitude explained 9.00% of the variance of distress [*F*(3,352) = 12.14; *p* < 0.001], as presented in Table 2.

## Discussion

The results support the evidence that a positive leisure attitude is associated with higher positive psychological well-being (Adams et al., 2011). Lampinen et al. (2006) confirmed that mental well-being, in later life, is linked with leisure activities, as our results revealed.

The main findings of the present study are that old-aged people with higher education levels show a better psychological well-being and a more positive leisure attitude, probably as a result of an indirect effect on the first variable. This outcome is in line with the results found by Tsai et al. (2014). They showed a significant relation between leisure attitude and mental health management. In addition, our findings are in accordance with the study of Chang et al. (2014), which showed that a positive attitude of involvement in leisure activities is associated with better health in older age. The results are consistent with the conclusions of other studies, once it was demonstrated that a positive attitude toward leisure activities helps to decrease the risk of mental health diseases (Morita et al., 2007; Teychenne et al., 2008; Rodríguez-Rodríguez et al., 2018). The results reached confirmed that a leisure attitude mediated the association between education and psychological well-being in this age group, as expected. Concerning the relation between distress and education, international research confirmed that a higher level of education was associated with lower levels of distress (Bossé et al., 1987; Ross and Zhang, 2008; Zhang et al., 2015; Amagasa et al., 2017). In this sense, a better mental health, understood as a combination of better psychological well-being and a lower distress level, was related with a higher education level in our sample. In addition, the study of Rosness et al. (2016) concluded that distress is higher in old people with less education. The same result was observed in Shivakumar et al. (2015) where illiteracy was considered a factor of distress in the sample studied.

However, an important finding was detected in our study which is not in line with standard results: the highest level of distress was found in participants with the highest level of education (as seen in Table 2); maybe a high level of education can lead to a better perception of all aging changes (body, life, profession, etc.). The transition to a new life-stage leads to several readjustments within the family and may disturb family functioning. This may enable people to perceive themselves as generally incapable of dealing with it and transform that incapacity into a stressor. Old-aged people have the need to adopt a positive leisure attitude to perceive themselves as useful, and this feeling may contribute to a stressful moment. This might be explained by the fact that “younger” old-aged people have more distress (as presented in Table 2). Thus, they recognize that a new life stage is coming and they have a good perception of personal and social changes as an essential aspect, and consider that a positive leisure attitude may be of extreme importance when preparing for retirement, as pointed out by Lee et al. (2018). Furthermore, it is important to evaluate needs, interests and expectations (Tsai et al., 2014), according to the different educational level of older adults.

## Conclusion

Aging represents the culmination of a long process of deliberation and discussion with contributions from various perspectives and scientific domains (Fernández-Ballesteros et al., 2004). It must be assumed as a positive experience, a new stage of life that is accompanied by changes and new routines. However, it’s assumed that aged people have a significant ongoing decline in physical capacities and cognitive function (Thomas et al., 2016). This worsening prompts their feelings of decreasing leisure autonomy competence as the range of their practically attainable achievements becomes limited in leisure activities (Chang and Yu, 2013). As showed by Lee et al. (2018), it is the way how individuals understand leisure and their beliefs about their ability to engage in leisure activities, that influence their orientation and attitude toward life.

The present study provides new information to understand the benefits of a positive leisure attitude in old adults. As the educational level is a variable which cannot be manipulated, leisure intervention programs shall consider the importance of a leisure attitude and contemplate strategies for stress reduction.

Future research should consider the indirect effect of gender as a moderated variable in the relation between education and mental health, mediated by leisure attitude. The study of significant differences between sub-samples of ages can help to understand aging in a longitudinal perspective. In addition, future research might consider a measure of different types of leisure activities to determine which ones influence more psychological well-being.

## Data Availability Statement

The datasets generated for this study will not be made publicly available. The subjects of our sample accepted to participate in this study but not that their data were published. Requests to access the datasets should be directed to the corresponding author.

## Ethics Statement

The study followed the ethical principles for research in humans, established in the Declaration of Helsinki in relation to informed consent, confidentiality and procedures. The Direction Boards of all the institutions approved the questionnaire and data collection. Participants received information about the research aims (doctoral study) before giving their consent to answer the questionnaire. They had the option to drop out the study at any time without giving any explanation. Data were processed as confidential and anonymized.

## Author Contributions

PB contributed substantially to the conception and design of the study and performed the analysis. PC and CM collected the data. RP conceived and designed the analysis. PB and EN-P drafted the manuscript. EN-P provided critical revision of the article. PB, EN-P, RP, PC, and CM provided final approval of the version to publish.

## Conflict of Interest

The authors declare that the research was conducted in the absence of any commercial or financial relationships that could be construed as a potential conflict of interest.
